# Combination therapy with GLP-1 receptor agonists and basal insulin: a systematic review of the literature

**DOI:** 10.1111/dom.12025

**Published:** 2012-11-12

**Authors:** R Balena, I E Hensley, S Miller, A H Barnett

**Affiliations:** 1 Eli Lilly and Company Ltd, Erl Wood ManorWindlesham Surrey, UK; 2 Eli Lilly and CompanyIndianapolis, IN, USA; 3 Amylin Pharmaceuticals, LLCSan Diego, CA, USA; 4 University of Birmingham and Diabetes Centre, Heart of England NHS Foundation TrustBirmingham, UK

**Keywords:** GLP-1, glycaemic control, insulin therapy, type 2 diabetes

## Abstract

Treatment algorithms for type 2 diabetes call for intensification of therapy over time as the disease progresses and glycaemic control worsens. If diet, exercise and oral antihyperglycaemic medications (OAMs) fail to maintain glycaemic control then basal insulin is added and ultimately prandial insulin may be required. However, such an intensification strategy carries risk of increased hypoglycaemia and weight gain, both of which are associated with worse long-term outcomes. An alternative strategy is to intensify therapy by the addition of a short-acting glucagon-like peptide-1 receptor agonist (GLP-1 RA) rather than prandial insulin. Short-acting GLP-1 RAs such as exenatide twice daily are particularly effective at reducing postprandial glucose while basal insulin has a greater effect on fasting glucose, providing a physiological rationale for this complementary approach. This review analyzes the latest randomized controlled clinical trials of insulin/GLP-1 RA combination therapy and examines results from ‘real-world’ use of the combinations as reported through observational and clinical practice studies. The most common finding across all types of studies was that combination therapy improved glycaemic control without weight gain or an increased risk of hypoglycaemia. Many studies reported weight loss and a reduction in insulin use when a GLP-1 RA was added to existing insulin therapy. Overall, the relative degree of benefit to glycaemic control and weight was influenced by the insulin titration employed in conjunction with the GLP-1 RA. The greatest glycaemic benefits were observed in studies with structured titration of insulin to glycaemic targets while the greatest weight benefits were observed in studies with a protocol-specified focus on insulin sparing. The adverse event profile of GLP-1 RAs in the reviewed trials was similar to that reported with GLP-1 RAs as monotherapy or in combination with OAMs with gastrointestinal events being the most commonly reported.

## Introduction

Type 2 diabetes is associated with overweight and obesity and has a complex pathophysiology characterized by abnormalities in insulin secretion, excess hepatic glucose production and insulin resistance in the liver and peripheral target tissues. As type 2 diabetes progresses, attaining and maintaining glycaemic control becomes increasingly challenging, risk of cardiovascular comorbidities increases, and weight gain is common 1. In turn, weight gain further worsens hyperglycaemia, hyperinsulinemia, insulin resistance and dyslipidemia.

Current treatment of type 2 diabetes begins with diet and lifestyle modification accompanied by use of a single oral antihyperglycaemic medication (OAM). As glycaemic control worsens, a second or third OAM is added. Ultimately, a basal insulin (such as neutral protamine Hagedorn insulin, insulin detemir or insulin glargine), or premixed insulin, and/or a prandial insulin, may be added to the treatment regimen 2. Despite, the addition of therapeutic agents and intensification of doses over time, glycaemic control often continues to deteriorate 2–6. A substantial proportion of patients with type 2 diabetes (50-60%) can achieve glycaemic targets by initiating basal insulin and using a structured dose titration regimen 7. However, in patients with long-standing type 2 diabetes, use of a prandial insulin is often required to maintain glycaemic control 2,8. Such intensification of insulin regimens increases the risk of hypoglycaemia and may lead to weight gain which can increase cardiovascular risk and worsen weight-related comorbidities 1. Hypoglycaemia is associated with increased mortality, risk of microvascular and macrovascular events and other adverse events 9,10. Importantly, hypoglycaemia also produces psychological distress and avoidance of hypoglycaemia can be a barrier to effective management of diabetes medications 11.

Glucagon-like peptide-1 receptor agonists (GLP-1 RAs), more recent therapeutic options in the type 2 diabetes treatment arsenal, improve glycaemic control while producing weight loss or maintenance without increasing the risk of hypoglycaemia when used alone 12–20. The first marketed GLP-1 RA was a short-acting twice daily (BID) formulation of exenatide approved for use in the United States in 2005 and in the European Union in 2006. Subsequent marketed GLP-1 RAs include liraglutide, a longer acting agent administered once daily (QD), and a long-acting formulation of exenatide administered once weekly (QW). Additional GLP-1 RAs are in clinical development, including another QD agent, lixisenatide and two QW agents, albiglutide and dulaglutide 21. GLP-1 RAs improve glycaemic control through multiple mechanisms of action including enhancement of glucose-dependent insulin secretion from pancreatic β-cells, glucose-dependent suppression of inappropriately elevated glucagon secretion, slowing the rate of gastric emptying and the absorption of meal-derived glucose, and reducing caloric intake 19–23. Deterioration of β-cell function over time is a primary reason that glycaemic control becomes increasingly challenging with the progression of type 2 diabetes 24 and some GLP-1 RAs also show promise in preserving and improving markers of β-cell function as evidenced by restoration of first-phase insulin secretion and enhancement of insulin synthesis and processing 19,20,25–27. In addition, Bunck et al. suggested that long-term exenatide BID treatment (3 years) produced a small, durable improvement in β-cell function which persisted 4 weeks after discontinuation of exenatide BID 27.

Effects of GLP-1 RA on blood glucose are believed to be mediated primarily through GLP-1 receptors on pancreatic islet cells, stomach, liver and brain 28. GLP-1 receptors have also been found in heart, kidneys and blood vessels, suggesting activation of these receptors may have direct effects on cardiovascular and other functions 29. Indeed many studies have observed that GLP-1 RA treatment is associated with favourable changes in risk factors or markers for cardiovascular disease such as blood pressure, triglycerides, low density lipoprotein cholesterol (LDL-C), C-reactive protein and adiponectin, as reviewed 29.

For patients who are unable to achieve adequate glycaemic control with basal insulin and OAMs, intensification of therapy with the addition of a short-acting GLP-1 RA may offer a number of advantages compared to the addition of rapid-acting prandial insulin. These include reduced risk of severe hypoglycaemia and weight gain compared to rapid-acting insulin, a mitigation of the weight gain associated with basal insulin therapy, and a reduced regimen complexity 30. The combination of basal insulin with a short-acting GLP-1 RA offers the advantage of complementary pharmacological properties resulting in improvement of both fasting and postprandial glycaemic control, respectively 14,30 (Table 1). Basal insulin controls fasting and preprandial glycaemia primarily by suppressing hepatic glucose production 2. In contrast, GLP-1 RAs reduce postprandial glucose excursions by slowing gastric emptying, reducing postprandial glucagon secretion and stimulating glucose-dependent insulin secretion. Lesser effects on postprandial glucose excursions have been observed with longer acting GLP-1 RAs such as liraglutide QD and exenatide QW 15,17. GLP-1 RAs also promote satiety, decrease food intake and reduce body weight 19,20,22,23,31,32.

**Table 1:** Complementary features of basal insulin and GLP-1 receptor agonists

	Basal insulin	GLP-1 receptor agonist
Primary effects	↓Fasting glucose	↓Postprandial glucose excursions
	↓Interprandial glucose	↓Fasting glucose*
Mechanism	↓Hepatic glucose production	↑Glucose-dependent insulin secretion
	↑Non-glucose dependent endogenous insulin	↓Glucagon secretion
	↓Glucagon secretion	↓Hepatic glucose production
	↑Insulin concentration	
		↓Gastric emptying rate
		↑Satiety
		↓Food intake
Effect on weight	↑Body weight	↓Body weight

* The most salient effect of GLP-1 RAs is on postprandial glucose, however, fasting glucose is also reduced, especially with longer acting GLP-1 RAs such as liraglutide and exenatide once weekly.

The use of exenatide BID as an add-on to insulin glargine, a long-acting basal insulin, was recently approved by the United States Food and Drug Administration (US FDA) and use of exenatide BID as an add-on to basal insulin was recently approved by the European Medicines Agency 33. The addition of insulin detemir in patients not achieving adequate glycaemic control with liraglutide QD was recently approved by the US FDA and the European Medicines Agency 34,35. For the new once daily GLP-1 RA lixisenatide, proposed use in combination with basal insulin is included the marketing authorization application filed with the European Medicines Agency in November 2011.

The objective of this review is to present results on all GLP-1 RAs that have been studied in combination with basal insulin and provide a clinical appraisal of safety and efficacy of these combinations. Focusing on studies in which patients were treated for more than 4 months, this review includes ‘real world’ evidence such as observational and clinical practice studies as well as randomized controlled clinical trials. Compared to earlier exenatide-focused reviews 36,37 here we provide an updated analysis of the most recent studies, including those using combinations of insulin with liraglutide QD or lixisenatide QD.

## Literature Search Methodology

A literature search was conducted in the following databases for the period of January 1, 2005 through December 31, 2011: Medline, Embase, Biosis and Current Contents. In addition, abstracts for presentations from the American Diabetes Association, European Association for the Study of Diabetes, and International Diabetes Federation annual scientific congresses occurring in 2011 were reviewed to identify references that had not yet been indexed in these databases. Review articles, preclinical studies, pharmacokinetic/pharmacodynamic studies, case studies and publications without safety or efficacy data for the combined use of insulin and GLP-1 RA combination were excluded. Both prospective and retrospective observational studies, clinical practice studies and controlled clinical trials in which patients were treated for more than 4 months were considered. Studies were included only if 30 or more patients were reported to have received combined insulin and GLP-1 RA therapy and if HbA1c change was reported. Studies appearing in abstract form may have been included if a manuscript was not yet available. Key efficacy and safety results were summarized based on the following parameters: glycaemic control (assessed with HbA1c, fasting glucose and postprandial glucose), body weight, insulin dose, rate/incidence of hypoglycaemia and overall safety profile. Ongoing trials of combined insulin and GLP-1 RA therapy were identified using the US National Institutes Health Clinical Trials Registry (http://www.clinicaltrials.gov). While the manuscript was under review three studies which were originally cited in abstract form appeared as published manuscripts and their citations were updated accordingly although the publication date was outside of the originally specified literature search window.

## Literature Identified

Manuscripts and abstracts from a total of 14 observational/clinical practice studies (Table 2) and five clinical trials (Table 3) met the criteria for inclusion in this review. Published information was available for the combined use of insulin with the GLP-1 RAs exenatide BID, liraglutide QD and lixisenatide QD. There were no studies of combination use of insulin and exenatide QW. The duration of combined GLP-1 RA and insulin therapy ranged from approximately 5–48 months across studies and thus provided a sufficient treatment period for establishing efficacy and assessing adverse events.

**Table 2:** Key efficacy results for clinical practice studies and observational studies examining combination therapy of GLP-1 receptor agonists and basal insulin in type 2 diabetes

				HbA1c (%)		Body Wt (kg)‖		Δ Insulin dose (total daily)	
						
References	Study type	Treatment duration	Treatment regimen	BL	Δ	BL	Δ	BL	Δ
Thong et al. 43	P Obs	Median 26 weeks	EXEN → Ins ± MET ± SU ± TZD§ (N = 1257)	9.55	↓0.51	112.7	↓5.8	120 U	↓42 U
		Median 27 weeks	<Control > EXEN without Ins ± MET ± SU ± TZD§ (N = 2936)	9.42	↓0.94	114.1	↓5.5	NA	
Pawaskar et al. 44	R Obs	Mean 12 months	EXEN → BasalIns (N = 1320)BasalIns → EXEN (N = 432)	8.5	↓0.5	111.4	↓4.0	NR	
Levin et al. 46	R Obs	24 months	InsGlar → EXEN ± MET ± SU ± TZD (N = 44)	8.9	↓1.0	112.2	↑0.7	NA	
			EXEN → InsGlar ± MET ± SU ± TZD (N = 121)	8.7	↓0.7	108.4	↓2.5	0.37 U/kg	↑0.10 U/kg
Sheffield et al. 16	R Obs	mean 14.6 months	EXEN → Ins (N = 134)	8.39	↓0.87	111.1	↓5.2	63 U (all) 48 U (basal)26 U (bolus)	↓5 U (all) ↑1 U (basal)↓9 U (bolus)
Levin et al. 45	R Obs	12 months	InsGlar → EXEN (N = 141)	8.9	↓0.9	NR		NA	
			EXEN → InsGlar (N = 281)	8.4	↓0.4	NR		NR	
Yoon et al. 41*	R Obs	mean 50 weeks	EXEN → Ins ± MET ± TZD ± SU ± α-glucosidase inhibitor ± meglitinide (N = 188)	8.05	↓0.54	117.8	↓5.5	99.9 U (all) 62.9 U (basal)29.4 U (prandial)	↓5.4 U (all) ↑ ∼ 4.5 U (basal)↓ ∼ 16.5 U (prandial)
Nayak et al. 47	P Obs	∼6 months	EXEN → Ins + MET (N = 160)	8.8	↓0.2	121.8	↓10.7	144 U	↓93 U
Viswanathan et al. 42	R Obs	mean 26 weeks	EXEN → Ins ± OAM(s) (N = 38)	7.7	↓0.6	116.4	↓6.4	58.4 U (basal) 50.4 U (rapid)72.9 U (mix)	↓5.3 U (basal) ↓13.8 U (rapid)↓44.6 U (mix)
Phillips et al. 48	CP	6 months	EXEN → MET + SU + InsGlar (N = 50)	8.2	↓1.4	133.6	↑4.1	105 U	↓8 U
Anholm et al. 39	CP	Mean 6.4 months	LIRA → Ins† ± MET ± SU (N = 115)	8.6	↓0.8	107.7	↓5.1	69 U	↓28 U
		Mean 7 months	LIRA → MET ± SU ± DPP-4 (N = 152)	8.7	↓1.4	106.4	↓3.5	NA	
Christofides et al. 64¶	CP	≤21 months	EXEN → Ins ± MET ± PIO (N = 109)	8.1	↓0.78	NR	↓4.3	NR	
			EXEN → MET ± PIO (N = 132)	7.1	↓0.79	NR	↓1.7	NA	
Houser et al. 79¶	CP	48 months	EXEN → Ins ± MET ± PIO (N = 47)	8.1	↓1.16	NR	↓7.3	NR	
			EXEN → MET ± PIO (N = 50)	7.1	↓1.06	NR	↓6.8	NA	
Vithian et al. 49	CP	Mean 19 weeks	EXEN → Ins ± OAM (unspecified) (N = 42)	8.9	↓0.75	NR	↓5.41%	NR	
Rachabattula et al. 61	R Obs	12 months	EXEN → Ins ± MET (N = 101)	9.4	↓1.3	120.5	↓4.5	135 U	↓21 U
Lind et al. 40	R obs	Mean 7 months	LIRA (N = 40) or EXEN (N = 21) → Ins‡ ± MET ± SU	8.9	↓1.0	111.1	↓7.1	91.1 U	↓38.6 U

BasalIns, basal insulin; BL, baseline; CP, clinical practice; DPP-4, dipeptidyl peptidase 4 inhibitor; EXEN, exenatide twice daily formulation; Ins, insulin; InsGlar, insulin glargine; LIRA, liraglutide; MET, metformin; NA, not applicable; NR, not reported; PIO, pioglitazone; P Obs, prospective observational; R Obs, retrospective observational; SU, sulphonylurea; TZD, thiazolidinedione.

* Efficacy results represent 18 to 27 months.

† 27% of patients were using InsGlar, and 35% were using premix insulin.

‡ 52.5% of patients received multiple daily injections, with 34% receiving basal and 11.5% receiving premix insulin.

§ Other OAMs were used, but frequency was low.

¶ These studies present data for the same patient population followed for different lengths of time.

‖ Body weight changes are reported in kg unless otherwise specified. Insulin doses reflect total daily doses unless otherwise specified.

**Table 3:** Key efficacy results for randomized controlled clinical trials examining combination therapy of GLP-1 receptor agonists and basal insulin in type 2 diabetes

				HbA1c (%)		Body Wt (kg)		Δ Insulin dose (total daily)	
						
Citation	Treatment duration	Background treatment*	Randomly assigned treatment	BL	Δ	BL	Δ	BL	Δ
**GLP-1 receptor agonist added to insulin**									
Buse et al. 52	30 weeks	InsGlar ± MET ± PIO	EXEN (N = 138)	8.32	↓1.7	95.4	↓1.8	49.5 U	↑13 U
			PBO (N = 123)	8.50	↓1.0	93.4	↑1.0	47.4 U	↑20 U
Seino et al. 53	24 weeks	SU + BasalIns†	LIXI (N = 154)	8.54	↓0.77	65.9	↓0.4	24.9 U	↓1.39 U
			PBO (N = 157)	8.52	↑0.11	65.6	↑0.1	24.1 U	↓0.11 U
**Insulin added to GLP-1 receptor agonist**									
Riddle et al. 54	24 weeks	MET + EXEN	EXEN + InsGlar (N = 17)	7.8	↓1.35	NR	↑0.4	NR	0.50 U/kg§
			PBO‡ + InsGlar (N = 17)		↓0.5	NR	↑4.1	NR	0.56 U/kg§
Blevins et al. 55	24 weeks	EXEN + MET ± SU or	InsGlar (N = 168)	8.2	↓1.4	102.3	↑0.7	NR	38 U§
Arakaki et al. 56		EXEN + MET ± PIO	ILPS (N = 171)	8.2	↓1.2	101.6	↑0.3	NR	31 U§
DeVries et al. 57¶	26 weeks	MET + LIRA	InsDet (N = 162)	7.6	↓0.5	96.0	↓0.2	NA	
			<Control > (N = 161)		↑0.02	95.3	↓1.0		
Bain et al. 58¶	52 weeks	MET + LIRA	InsDet (N = 130)	7.6	↓0.5	NR	↓0.1	NA	
			<Control > (N = 92)		↑0.01	NR	↓1.0		

BasalIns, basal insulin; BL, baseline; EXEN, exenatide twice daily formulation; ILPS, insulin lispro protamine suspension; InsDet, insulin detemir; InsGlar, insulin glargine; LIRA, liraglutide; LIXI, lixisenatide; MET, metformin; NA, not applicable; NR, not reported; PBO, placebo; PIO, pioglitazone; SU, sulphonylurea.

* Does not include treatments that were discontinued prior to start of randomly assigned treatment.

† Basal insulins were InsGlar (60%), InsDet (27%), neutral protamine Hagedorn insulin (13%); <1% also used premix insulin.

‡ Exenatide was replaced with placebo.

§ Insulin dose at 24 weeks.

¶ DeVries et al. 57 and Bain et al. 58 present analyses at different time points for the same study.

Approximately 5000 patients were reported to have received combination treatment with a GLP-1 RA and insulin. The majority of GLP-1 RA exposures (approximately 90%) were among patients treated with exenatide BID and were reported in observational studies. This finding is as expected given that exenatide BID received marketing authorization 5 years before liraglutide. Nonetheless, recent audits in the UK indicate that approximately 40% of 2303 patients treated with liraglutide use the agent in combination with insulin 38. As lixisenatide QD is still in development, all data for this GLP-1 RA in combination with insulin were limited to clinical trials.

Across the publications, the average duration of diabetes in study subjects ranged from 7 to 15 years. Single or dual use of concomitant OAMs in combination with insulin was reported in all clinical trials and in most of the observational and clinical practice studies. OAMs used, in decreasing frequency, were metformin, sulphonylureas, thiazolidinediones, and DPP-4 inhibitors.

In clinical trials, GLP-1 RAs were only used in combination with long-acting basal insulins (insulin glargine, insulin lispro protamine suspension [ILPS], or insulin detemir). In observational/clinical practice studies, insulin type was not always identified. When it was identified, basal insulin, particularly insulin glargine, was cited most frequently. Small numbers of patients received multiple daily injections of insulin (premixed insulin or basal insulin plus short-acting insulin or rapid-acting insulin) in observational/clinical practice studies 39–42. In none of these studies did it appear that GLP-1 RA was administered as a substitute for existing prandial insulin.

Across studies it was more common to find short-acting GLP-1 RA treatment added to an existing insulin regimen (with or without concomitant OAMs) rather than insulin added to existing GLP-1 RA therapy. Of the 19 studies reviewed, only 3 clinical trials and 2 observational studies (approximately 657 total patient exposures) described clinical outcomes for the addition of insulin to existing GLP-1 RA therapy.

## Clinical Efficacy

### HbA1c, Body Weight and Insulin Dose

#### Observational/Clinical Practice Studies

**Figure 1:**
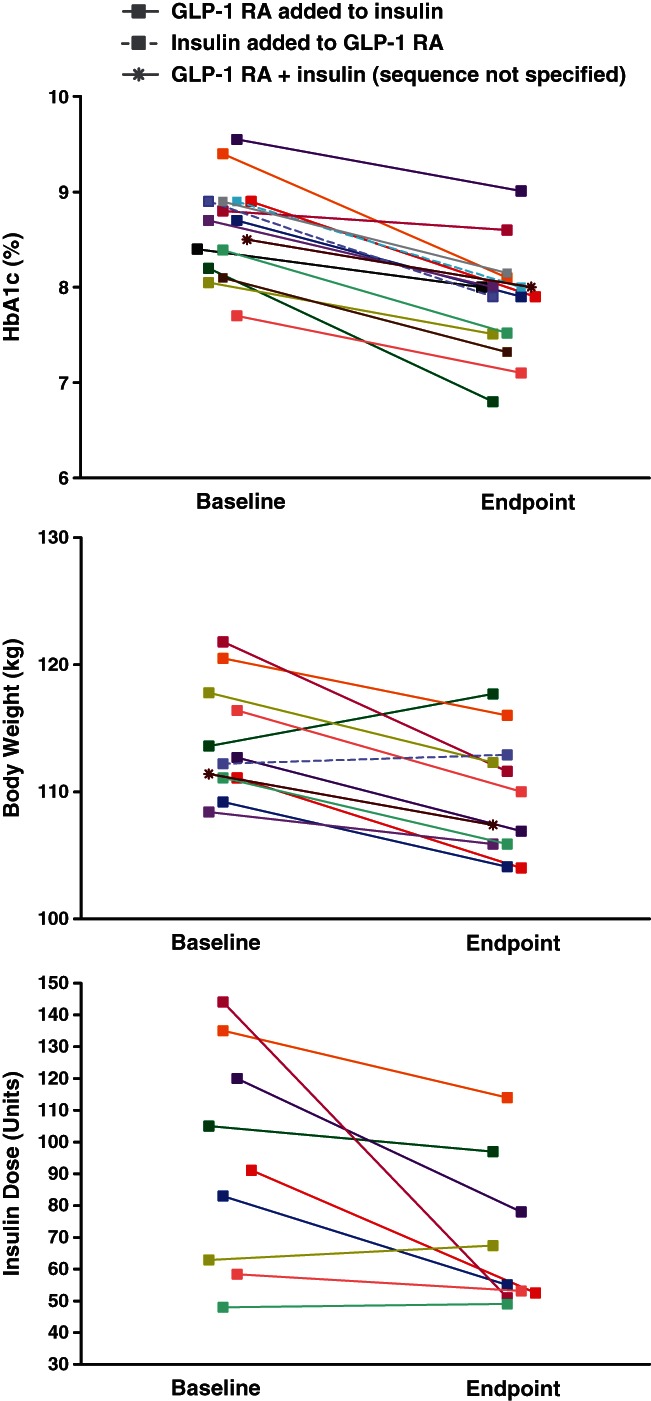
Key efficacy results from observational studies of combination therapy with insulin and a GLP-1 receptor agonist. Colour coding indicates changes in (A) HbA1c (B) body weight and (C) insulin dose between baseline and endpoint. Note, not all parameters were reported for each study.

A number of real world observational studies have reported the potential beneficial effects of the short-acting GLP-1 RA/insulin combination on both glucose control (HbA1c) and body weight reduction (Table 2). All studies identified in the literature review reported HbA1c as a parameter of glycaemic control, almost all studies reported body weight, and many reported changes in insulin use. Data on other parameters of glycaemic control (fasting and postprandial glucose) were reported infrequently. Changes in HbA1c, body weight and insulin dose with combined GLP-1 RA and insulin treatment in observational studies are illustrated in Figure 1 with a line representing each published report.

The largest analysis to date of combined exenatide and insulin treatment is a nationwide audit of exenatide BID use conducted by the Association of British Clinical Diabetologists (ABCD) 43. Their report evaluated the prevalence of combined use of exenatide BID with any type of insulin and outcomes associated with long-term use of the combination. Median patient follow-up was more than 6 months and outcomes were compared with those of patients using exenatide BID without insulin. Of 4857 patients using exenatide BID who had baseline and follow-up data, 1257 patients added exenatide to existing insulin therapy, 664 patients added insulin to existing exenatide therapy, and 2936 patients used exenatide without insulin. There was no insulin only comparison group and types of insulin used were not specified. Patients on exenatide BID alone experienced a 0.94% reduction in HbA1c and a body weight reduction of 5.5 kg. Patients already on insulin who added exenatide also experienced reductions in HbA1c and body weight (0.51% and 5.8 kg, respectively) while reducing insulin use from 120 U/day at baseline to 78 U/day at the end of follow-up.

In another large study, Pawaskar et al. reviewed patients from the General Electric Electronic Medical Record Database who initiated insulin treatment with insulin glargine, insulin detemir or the intermediate-acting insulin, neutral protamine Hagedorn within 6 months before or after initiating exenatide BID treatment 44. Approximately 25% of patients (432 of 1752) added insulin to exenatide BID with the remaining majority of patients (1320) adding exenatide BID to insulin. Over the 12-month post-index period HbA1c decreased from 8.5 to 8.0% and body weight decreased by 4 kg from a baseline of 111 kg. In a retrospective insurance claims database study, Levin et al. observed similar HbA1c improvements over a 12-month period for patients receiving each GLP-1 RA/insulin treatment sequence 45.

Results from a number of retrospective medical chart reviews are consistent with the large studies described above. For example, Sheffield et al. conducted a clinical case review of 134 patients who received exenatide BID and insulin (basal and/or rapid-acting insulin analogues) 16. Patients experienced a mean HbA1c reduction of 0.87% after a mean of 14.6 months and the proportion of patients achieving HbA1c <7% increased to 37 from 16% at baseline. Body weight showed mean 5.2 kg reduction similar to that reported in other studies. Importantly, while neither the mean total daily insulin dose nor the mean basal insulin dose was significantly changed in patients who received the combined treatment, 45% of patients using rapid-acting insulin were able to discontinue the medication and 35% reduced their rapid-acting insulin dose. The proportion of patients using sulphonylureas and mean sulphonylurea dose also declined.

Findings from a chart review by Levin et al. 46 were consistent with the ABCD study. They evaluated 165 patients treated for up to 24 months with the combination of insulin glargine and exenatide BID. Regardless of the sequence in which the medications were added [(glargine to exenatide (n = 44) or exenatide to glargine (n = 121)], HbA1c was significantly reduced at 24 months. For patients adding insulin glargine to exenatide BID, HbA1c was reduced by 1% from a baseline of 8.9% while those patients adding exenatide BID to insulin glargine experienced a mean HbA1c reduction of 0.7% from a baseline of 8.7%. By study endpoint, 34% of patients who had added exenatide BID to insulin glargine had achieved an HbA1c ≤7%, a therapeutic target consistent with ADA guidelines, while 31% of patients in the other treatment sequence also achieved the same goal. Body weight was significantly reduced in the exenatide BID added to insulin glargine group (−2.5 ± 6.7 kg) but was not significantly changed in the group that added insulin glargine to exenatide BID (+0.7 ± 8.3 kg). Likewise, Yoon et al. made similar observations in a retrospective clinical practice review which evaluated 188 patients adding exenatide BID to insulin therapy (basal with or without prandial) 41. At 18–27 months, mean reduction in HbA1c was 0.5% and mean weight reduction was 5.5 kg.

Progressive weight gain associated with insulin therapy is a major challenge for diabetes patients, particularly those with obesity. Several studies focused on the use of exenatide BID and insulin (basal with or without prandial) combination therapy in patients with obesity and type 2 diabetes 42,47–49. For example, Nayak et al. undertook a prospective study in 174 consecutive obese patients (mean BMI 43 kg/m^2^) with type 2 diabetes and poor glycaemic control (mean HbA1c 8.8%) 47. The initial focus of the protocol (0–6 months) was to promote weight loss by maximizing metformin utilization, introducing exenatide BID and down-titrating insulin dosages while maintaining or improving glycaemic control. At 6–12 months the protocol focus shifted to maintaining weight loss and correcting any residual problems with glycaemic control. The types of insulin used were not specified. In the 160 patients who completed 6 months of therapy, mean weight loss was approximately 11 kg, total daily insulin dose decreased from 144 to 51 U/day, and HbA1c was not significantly changed (8.8% at baseline, 8.6% at 6 months). In the 57 patients who completed 12 months of treatment, mean reductions in weight (13 kg from baseline) and insulin use (159 U/day at baseline, 55 U/day at 12 months) were maintained but HbA1c did not improve (9.2% at baseline, 9.1% at 12 months). The lack of improvement in HbA1c was ascribed by the investigators to the inertia both from patients and physicians to up-titrate insulin because of apprehension that it would slow weight loss or induce regain of body weight. It is well established that this population with severe obesity and poor glycaemic control is challenging to manage successfully due to the risk of aggravating their obesity with intensification of insulin therapy 50,51.

In another study with obese patients, Viswanathan et al. conducted a retrospective chart review of 52 patients with type 2 diabetes in whom exenatide BID was added to insulin with a mean follow-up period of 26 weeks 42. Insulins used included rapid-acting, mixed and basal. They observed a mean body weight reduction of approximately 6.5 kg, a mean HbA1c decrease of 0.6%, and significant reductions in rapid-acting and mixed insulin dosages. In an abstract on a clinical practice study of 50 obese patients with type 2 diabetes, Phillips et al. reported that addition of exenatide BID to existing therapy with metformin, glipizide and insulin glargine led to a significant reduction in HbA1c from 8.2% at baseline to 6.8% for the 35 patients completing 6 months of treatment 48. In contrast to other reports on combined GLP-1 RA and insulin treatment, there was an increase in body weight from 134 to 138 kg, although it was not statistically significant. Insulin glargine was infrequently titrated (6–8 week intervals) in this study and insulin dose was not significantly reduced during the course of the trial which may be related to the lack of weight benefit from the combination.

Due to the design of most of the observational studies the insulin titration schemes used were unknown and likely highly variable; however, as expected, the studies with the largest reductions in weight (reductions of 5–6 kg) also reported significant reductions in prandial insulin dose, total daily insulin dose, and/or percentage of patients using prandial insulin 16,41–43,47. The two studies of exenatide BID plus insulin combination therapy which had a protocol-specified focus on insulin sparing reported very favourable changes in weight (6–10 kg) but modest improvements in glycaemic control (0.2–0.6% HbA1c reductions) 42,47. In contrast the study which reported limited down-titration of insulin had substantial gains in glycaemic control (HbA1c, −1.4%) and no improvement in body weight following the addition of exenatide BID to insulin glargine therapy 48.

Other observational and clinical practice studies (Table 2) reported HbA1c, body weight and insulin improvement similar to most of those described above. One of the most consistent observations across these studies was the notable reduction in body weight when a GLP-1 RA was used in combination with insulin. GLP-1 RAs have been shown to increase satiety and decrease food intake. In addition, changes in lifestyle associated with enthusiasm for starting a new therapy may have contributed to body weight reduction. These findings from observational and clinical practice studies show the potential benefits of GLP-1 RA for lowering HbA1c, body weight and total daily insulin dose when added to patients not adequately controlled on insulin therapy. However, there are several limitations that need to be acknowledged. These include the lack of a parallel comparator group, the absence of protocol-specified criteria to monitor changes in background medications, potential underreporting of adverse events (only those captured in the medical chart), and potential selection bias by the investigator in the review of medical records. The randomized controlled clinical trials that have examined the combination therapies are free from many of these limitations.

#### Randomized Controlled Trials

Combinations of GLP-1 RAs and insulin have also been evaluated in a number of recent randomized controlled clinical trials investigating exenatide BID, liraglutide QD and lixisenatide QD (Table 3).

##### GLP-1 receptor agonist added to insulin

Buse et al. conducted the first double-blind, placebo-controlled study of concomitant therapy with a GLP-1 RA (exenatide BID) added to existing basal insulin therapy (insulin glargine) 52. Patients on pre-existing (≥3 months) insulin glargine (with or without OAMs) were randomized to placebo (n = 123) or exenatide BID (n = 138) and insulin glargine was systematically titrated according to the Treat-to-Target algorithm 7 with the goal of achieving fasting plasma glucose below 5.6 mmol/l (100 mg/dl). Most patients had long-standing type 2 diabetes (mean duration was 12 years and approximately 14% of patients had diabetes duration ≥20 years). After 30 weeks of treatment with exenatide BID or placebo, the exenatide group exhibited decreases in HbA1c and weight (mean changes from baseline −1.7% and −1.8 kg, respectively), whereas the placebo group had a smaller reduction in HbA1c (−1.0%) and gained weight (+0.96 kg). The difference in HbA1c levels between exenatide and placebo was similar regardless of OAM use and subject age. An important aspect of the design of the Buse et al. study was the aim to optimize insulin therapy through titration to fasting glucose targets. Although, mean insulin doses increased in both groups, the greater improvement in glycaemic control with exenatide BID was observed with a significantly smaller increase in daily insulin dose (13 vs. 20 U/day), with associated weight loss rather than weight gain, and with no increased risk of hypoglycaemia. Numerous methodological differences make comparisons difficult between trials, however, it should be noted that the reduction in HbA1c in this study was greater than in the exenatide plus insulin observational and clinical practice trials while the reduction in weight was more modest than in most of those trials. These differences may be explained by the systematic up-titration of insulin glargine to optimize glycaemic control in Buse et al. 52 while most of the observational and clinical practice trials reported decreased insulin use which often included both prandial and basal insulin. In addition, baseline weights were higher in the observational studies (108–134 kg) compared to this study (94 kg), suggesting a greater margin for improvement in weight.

In the only other available randomized controlled study of a GLP-1 RA added to basal insulin, Seino et al. reported on the efficacy and safety of 20 mcg lixisenatide QD versus placebo in 311 Asian patients with type 2 diabetes inadequately controlled with basal insulin (±sulphonylurea) over a treatment period of 24 weeks 53. Addition of lixisenatide led to a 0.8% reduction in HbA1c from a baseline of 8.5% with 36% of patients achieving HbA1c <7% and 18% achieving HbA1c ≤6.5%. Fasting glucose (−0.42 mmol/l) and insulin dose (−1.4 U/day) were modestly reduced, and there were non-significant decreases in body weight (−0.4 kg).

##### Insulin added to GLP-1 receptor agonist

Three randomized controlled trials have added basal insulin to existing GLP-1 RA therapy. Riddle et al. examined the addition of insulin glargine to metformin plus exenatide BID treatment (MEXELIN) 54. After an 8-week lead-in on metformin plus exenatide BID, patients were randomly assigned to receive either metformin/exenatide/insulin glargine (n = 17) or metformin/ placebo/insulin glargine (n = 17) for an additional 24 weeks. Insulin glargine was systematically titrated according to the Treat-to-Target algorithm 7. More patients achieved HbA1c targets <6.5% and <7.0% with metformin/exenatide/insulin glargine treatment (47% and 76%, respectively) compared to those receiving metformin/placebo/insulin glargine (12% and 24%, respectively). In contrast to the 4.1-kg weight gain that resulted with metformin/placebo/insulin glargine, there was minimal weight gain (0.4 kg) with metformin/exenatide/insulin glargine treatment.

A larger open-label, multicenter, randomized, 24-week clinical trial compared two basal insulin/exenatide BID combination therapies 55,56. Insulin glargine (n = 168) or ILPS (n = 171) was added to existing exenatide BID and OAMs. Basal insulin was systematically titrated to achieve fasting glucose below 5.5 mmol/l. HbA1c was reduced 1.2% with the ILPS combination and 1.4% with the insulin glargine combination and the percentage of patients achieving HbA1c <7.0% was not significantly different between treatments. Weight gain (<1 kg) was similar between treatments while the total insulin dose was lower for ILPS compared to insulin glargine (31 vs. 38 U/day). These results show that either insulin can be used to effectively improve glycaemic control in patients inadequately controlled with oral agents and exenatide BID.

Finally, DeVries et al. 2012 reported a triple therapy study where basal insulin detemir was added to liraglutide QD plus metformin 57. This liraglutide/insulin combination trial employed a similar order of therapy intensification as used in the MEXELIN trial. A 12-week lead-in with liraglutide plus metformin was followed by 26-week randomized period in which subjects not reaching HbA1c <7.0% during the lead-in either added insulin detemir or stayed on metformin plus liraglutide. Sixty-one percent of patients achieved HbA1c <7% during the lead-in period and had a mean HbA1c reduction of 1.3%. Patients not reaching HbA1c <7% during the lead-in had a mean HbA1c reduction of approximately 0.6%. From this group, patients randomly assigned to intensification of therapy with the addition of detemir for 26 weeks experienced a further HbA1c decrease of 0.5% while mean HbA1c was stable for those remaining on metformin plus liraglutide. Mean body weight decreased in all groups during the lead-in period (−3.5 to −4.4 kg) and remained stable or decreased further, even in the patients adding insulin detemir during the randomized period (−0.2 kg). HbA1c and weight remained stable in an extension covering an additional 26 weeks 58.

In the randomized controlled trials, addition of GLP-1 RA to existing basal insulin therapy resulted in improved glycaemic control with modest loss 52 or non-significant change in weight 53. In general, when basal insulin was added to existing GLP-1 RA therapy there was improvement in glycaemic control with no change in weight or minimal weight gain in both the randomized trials 54–56,58,59 and the observational studies 45,46.

### Postprandial Glucose

**Figure 2:**
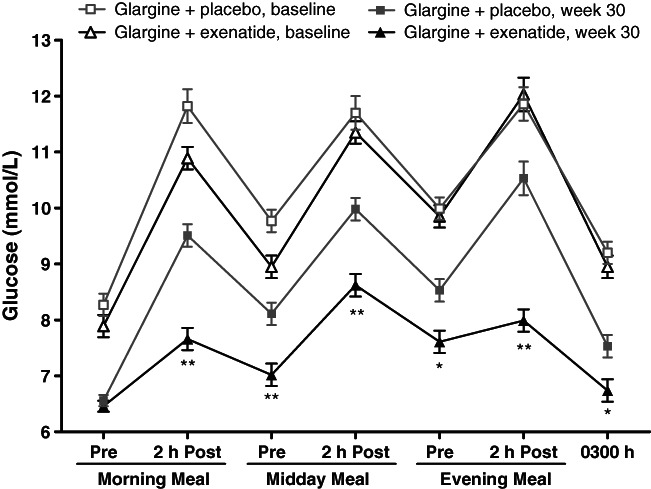
Self-monitored blood glucose concentration in patients receiving insulin glargine in combination with either placebo or exenatide twice daily for 30 weeks. Values are least-squares mean ± standard error. *p < 0.01, **p < 0.001 for between-group comparison. Adapted with permission from 52.

Two randomized controlled trials have described the changes in glucose excursions following meals when patients are treated with the combination of GLP-1 RA and insulin. Buse et al. 52 reported, based on self-monitored blood glucose, that at all time points (except the morning pre-meal/fasting) exenatide BID used in combination with insulin glargine was associated with both statistically and clinically significant lowering of blood glucose compared with placebo and insulin glargine (generally by ≥1 mmol/l; Figure 2). Furthermore, average postprandial glucose levels in patients treated with exenatide BID and insulin glargine were well within current treatment targets, particularly after the morning and evening meals, when exenatide BID was administered.

More recently, the addition of lixisenatide QD (20 mcg once daily) to basal insulin has been associated with significant improvement in 2-h postprandial glucose, glucose excursions and average 7-point self-monitored blood glucose which were reduced by a mean of 1.9 mmol/l (vs. 0.6 mmol/l reduction with placebo) 53.

No randomized trials have reported on the addition of liraglutide QD to basal insulin therapy, however, when exenatide BID and liraglutide QD were compared head-to-head (not in combination with insulin), exenatide BID was associated with greater improvement in post-prandial glucose than liraglutide QD (treatment group difference of 1.3 mmol/l after breakfast and 1.0 mmol/l after dinner) 17. In contrast, improvements from baseline in HbA1c and fasting plasma glucose were greater with liraglutide (treatment differences of 0.3% and 1.1 mmol/l, respectively). Given the paucity of data on prandial changes in patients treated with GLP-1 RAs in combination with insulin, head-to-head studies are needed to determine the relative benefits of each combination.

### Other Efficacy Parameters (Blood Pressure, Lipid Profile)

Type 2 diabetes is associated with increased risks of cardiovascular disease and many studies have shown improvements in cardiovascular risk factors with GLP-1 RA therapy 29. Here, we summarize what has been reported about cardiovascular changes associated with GLP-1 RAs when used in combination with insulin.

In controlled clinical trials decreases in blood pressure have been reported in two studies of GLP-1 RA/basal insulin combination therapy. Buse et al. 52 reported that 30 weeks of treatment with exenatide BID added to existing insulin glargine resulted in statistically significant decreases in systolic (−2.7 mm Hg) and diastolic (−1.7 mm Hg) blood pressure in contrast to increases in the group adding placebo to insulin glargine (+1.7 mm Hg systolic, +1.7 mm Hg diastolic). These improvements occurred in patients who were already controlled with concomitant drug therapy for hypertension. There were no significant differences between groups in triglycerides, high density lipoprotein cholesterol (HDL-C), LDL-C and non-HDL-C. Rodbard et al. 60 described blood pressure changes in patients adding insulin detemir for 26 weeks following a 12-week lead-in period on metformin and liraglutide QD. They reported decreases in systolic blood pressure (−1.6 to −3.3 mm Hg) and diastolic blood pressure (−0.9 to −1.3 mm Hg) during the lead-in followed by minimal non-significant differences after intensification of therapy with insulin detemir.

Available evidence from observational studies, case reviews and clinical practice studies on the changes in cardiovascular risk factors associated with GLP-1 RA and insulin therapy was limited to studies of combinations of insulin with exenatide BID. Pawaskar et al. 44 observed statistically significant reductions in several lipid parameters over 12 months in a large medical record database review: total cholesterol (−3.9 mg/dl, −0.1 mmol/l), triglycerides (−8.9 mg/dl, −0.1 mmol/l), LDL-C (−1.9 mg/dl, −0.05 mmol/l), non-HDL-C (−3.9 mg/dl, −0.1 mmol/l), and HDL-C (−0.8 mg/dl, −0.02 mmol/l). These changes were accompanied by a very small but statistically significant reduction in diastolic blood pressure from 75.6 mm Hg to 74.9 mm Hg. In a smaller case review study Sheffield et al. 16 observed statistically significant decreases in total cholesterol (−5 mg/dl, −0.13 mmol/l) and triglycerides (−6 mg/dl, −0.07 mmol/l) in patients receiving exenatide BID therapy combined with insulin. These reductions occurred while patients maintained their current number and dose of hydroxymethylglutaryl-coenzyme A (HMG CoA) reductase inhibitors. LDL-C and HDL-C were not changed significantly. Likewise, there were no significant changes in systolic or diastolic blood pressure which were both normal at baseline.

In their insurance claims database study, Levin et al. reviewed the lipid profile from 44–45 patients who added insulin glargine to existing exenatide BID treatment and 77–79 patients who used the opposite treatment sequence 45. When exenatide BID was added to insulin glargine, there was a statistically significant decrease in LDL-C (−11.4 mg/dl, −0.30 mmol/l) and a decrease in triglycerides (−14.8 mg/dl, −0.17 mmol/l) which was not statistically significant. Triglyceride reduction was statistically significant when insulin glargine was added to exenatide BID (−60.0 mg/dl, −0.68 mmol/l). In a separate retrospective chart review, Levin et al. found no significant changes in blood pressure over 24 months of follow up in patients who combined GLP-1 RA and insulin in either sequence 46.

Beneficial changes associated with the combination therapy were also reported from observational and clinical practice studies focused on type 2 diabetes patients with obesity. Nayak et al. reported a significant reduction in systolic blood pressure in patients treated with exenatide BID and insulin (−9 mm Hg at 12 months compared to baseline) 47 while diastolic blood pressure increased slightly (+4 mm Hg at 12 months) and total cholesterol was unchanged. Viswanathan et al. reported a marked 26% decrease in plasma triglycerides (−53 mg/dl, −0.60 mmol/l) in patients who added exenatide BID to insulin therapy 42. The reduction in total cholesterol (−14 mg/dl, −0.36 mmol/l) was statistically significant but similar to that in the control group and no significant changes were observed in HDL-C or LDL-C. Notably, total cholesterol was within the normal range at baseline and all patients were taking HMG CoA reductase inhibitors which remained at stable doses throughout treatment. Systolic blood pressure was decreased by 9 mm Hg in patients receiving exenatide BID and insulin while diastolic pressure did not change significantly in either treatment group. Finally, in a small clinical practice study with obese patients, Phillips et al. 48 reported significant reductions of in triglycerides (−63 mg/dl, −0.71 mmol/l) and total cholesterol (−19 mg/dl, −0.21 mmol/l) which were of similar magnitude as in the Viswanathan et al. study 42. Smaller reductions in HDL and LDL-C did not reach statistical significance.

In general, results from these real-world studies are consistent with results from controlled clinical trials evaluating exenatide BID (not in combination with insulin) relative to placebo or insulin. In those studies reductions in systolic blood pressure and triglycerides were the most consistent findings 29.

## Adverse Events and Tolerability

Information on adverse events and tolerability for GLP-1 RAs used in combination with insulin is presented in Table 4. These data show a similar overall profile for combination treatment with GLP-1 RA and insulin as has been previously reported for use of GLP-1 RAs as monotherapy or in combination with OAMs. Furthermore, this literature review revealed no specific event or pattern of safety concerns to indicate a unique safety profile when GLP-1 RAs are combined with insulin therapy.

**Table 4:** Adverse events in studies examining combination therapy of GLP-1 receptor agonists and basal insulin in type 2 diabetes*

References	Treatment	SU?	BL dose adjustments	Hypoglycaemia	Severe hypo?	GI events	Notable events	DC adverse events
**Clinical practice and observational studies**								
Thong et al. 43	EXEN + Ins ± MET ± SU ± TZD† (N = 1921)	Yes	NR	8.9% (vs. 6.6% before added EXEN, hypoglycaemia)	<1%	28% (56% transient)	Acute renal failure (0.3%)	GI events: 36% Other AEs:15% NR
	<Control > EXEN ± MET ± SU ± TZD† (N = 2936)			6.1% (hypoglycaemia)	No	25% (76% transient)	Acute renal failure (0.2%)	GI events: 32% Other AEs:17%
Pawaskar et al. 62	EXEN + Ins (N = 472)	Yes	NR	Hypoglycaemia 1.9% (0.03 events/pt/6 months)	NR	NR	NR	NR
	Ins (N = 312)			2.9% (0.1 events/pt/6 months)				
	EXEN (N = 498)			1.8% (0.02 events/pt/6 months)				
Levin et al. 46	InsGlar + EXEN (N = 44)	Yes	NR	11%(hypoglycaemia) 203 days (time to first event) 57 mg/dl (mean glucose)	2%	NR	NR	NR
	EXEN + InsGlar (N = 121)			12% (hypoglycaemia) 229 days (time to first event) 52 mg/dl (mean glucose)	2%			
Sheffield et al. 16	EXEN + Ins (N = 134)	NR	NR	10% (hypoglycaemia)	<1%	Overall GI (42%) Vomiting (7%)	NR	GI events (20%): vomiting (5%); hypoglycaemia, itching, urinary retention, gastroparesis, cancer (<1%)
Levin et al. 45	InsGlar + EXEN (N = 141)	NR	NR	5.0% (hypoglycaemia, 6–12 months follow-up)	NR	NR	NR	NR
	EXEN + InsGlar (N = 281)			5.3% (hypoglycaemia, 6–12 months follow-up)				
Yoon et al. 41	EXEN + Ins (N = 188)	Yes	NR	4% (hypoglycaemia)	No	Nausea (23%); Vomiting (20%);Heartburn (1%);Diarrhoea,Constipation (<1%)	NR	26% ever treated with EXEN + Ins: nausea (16%); vomiting (8%); acute renal failure, constipation, generalized edema, heartburn, malaise, pancreatitis (<1%)
Nayak et al. 47	EXEN + Ins + Met (N = 160)	No	Maximize MET use; DC any SU	NR	No	NR	1 death (CV event)	GI events from 6–12 months: 5%
Viswanathan et al. 42	EXEN + Ins ± OAM(s) (N = 38)	NR	If HbA1c <7.5%, ↓Ins dose 10%	Rare (hypoglycaemia)	No	Mild, transient nausea	NR	Nausea (10%)
Phillips et al. 48	EXEN + InsGlar (N = 50)	Yes	NR	NR	No	NR	2 suspected pancreatitis cases	30%: nausea/vomiting (28%); chest pain (2%)
Vithian et al. 49	EXEN + Ins ± OAM (unspecified) (N = 42)	NR	NR	NR	NR	Nausea (35%)	NR	Nausea (5%); headache (2%)
Rachabattula et al. 61	EXEN + Ins (N = 101)	NR	NR	NR	NR	10%	NR	GI events (10%): nausea, vomiting, abdominal cramps
Lind et al. 40	LIRA (N = 40) or EXEN (N = 21) + Ins	Yes	NR	0.085 events/pt/last month below 70 mg/dl (asymptomatic) 0 below 52 mg/dl (asymptomatic) 0.24 events/pt/last month below 70 mg/dl (documented) 0.068 below 52 mg/dl; (documented)	2%	NR	1 death (myocardial infarct)	7%: Nausea (3%), myocardial infarct (2%), acute sepsis affecting liver (2%)
**Randomized controlled clinical trials**								
Buse et al. 52	EXEN + InsGlar (N = 138)	No	If HbA1c ≤8%, ↓InsGlar dose 20% until Week 5	25% (mild) 17% (mild nocturnal) 1.4 events/pt yr	No	Nausea (41%) Diarrhoea (18%) Vomiting (18%) Constipation (10%)	6% serious	9.5% (events not specified)
	PBO + InsGlar (N = 123)			29% (mild) 26% (mild nocturnal) 1.2 events/pt yr	<1%	Nausea (8%) Diarrhoea (8%) Vomiting (4%) Constipation (2%)	9% serious;1 death	<1% No data are available
Riddle et al. 54	EXEN + InsGlar (N = 17)	No	NR	53% (mild)	No	NR	NR	NR
	PBO + InsGlar (N = 17)			41% (mild)	No			
Seino et al. 53	LIXI + BasalIns (N = 154)	Yes	NR	47% (symptomatic, SU) 33% (symptomatic, no SU)	No	Nausea (40%) Vomiting (18%) Adb.discomfort (7%) Diarrhoea (7%)	7% serious	9% (mostly GI)
	PBO + BasalIns (N = 157)			22% (symptomatic, SU) 28% (symptomatic, no SU)	No	Nausea (5%) Vomiting (2%) Adb.discomfort (0.6%) Diarrhoea (3%)	6% serious;1 death	3%
Blevins et al. 55	EXEN + InsGlar (N = 168)	Yes	NR	18.1 events/pt yr (overall) 3.0 events/pt yr (nocturnal)	No	NR	NR	NR
Arakaki et al. 56	EXEN + ILPS (N = 171)			16.3 events/pt yr (overall), 4.9 events/pt yr (nocturnal)	2%			
DeVries et al. 57	LIRA + InsDet (N = 163)	No	DC any SU	0.29 events/pt yr (minor)	No	Nausea (17%) Diarrhoea (17%) Vomiting (9%)	5.5% serious	2.5% (events not specified)
	<Control > LIRA (N = 159)			0.03 events/pt yr (minor)	No	Nausea (23%) Diarrhoea (15%) Vomiting (10%)	3.8% serious; Pancreatitis:1 acute, 1 chronic	3.7% (events not specified)
Bain et al. 58	LIRA + InsDet (N = 130)	No	DC any SU	0.23 events/pt yr (minor)	No	NR	NR	NR
	<Control > LIRA (N = 92)			0.03 events/pt yr (minor)	No			

BL, baseline; CV, cardiovascular; DC, discontinuation; EXEN, exenatide twice daily formulation; GI, gastrointestinal; Hypo, hypoglycaemia; ILPS, insulin lispro protamine suspension; Ins, insulin; InsDet, insulin detemir; InsGlar, insulin glargine; LIRA, liraglutide QD; LIXI, lixisenatide QD; MET, metformin; NR, not reported; OAM, oral antidiabetes medication; PBO, placebo; pt yr, patient year; SU, sulphonylurea; TZD, thiazolidinedione.

* Unless otherwise specified, percentages are percentage of patients experiencing event.

† Other OAMs were used, but frequency was low.

### Overall Adverse Events Profile

Gastrointestinal events were consistently reported across the literature reviewed. Nausea was most common, affecting 23–41% of exenatide BID-treated patients 41,49,52, 40% of lixisenatide QD-treated patients 53, and 17–26% of liraglutide-treated patients 57. Vomiting and diarrhoea were also reported in some studies (Table 4). Gastrointestinal events were primarily considered mild and transient in nature. These events were also the most frequently cited type of adverse event that led patients to discontinue GLP-1 RA treatment 16,41,47,48,53,57,61. This event pattern is consistent with the literature for GLP-1 RAs used alone or in combination with OAMs showing that gastrointestinal events with GLP-1 RA therapy tend to be mild or moderate in severity and decrease in occurrence with continued dosing 19,20,23.

Non-gastrointestinal adverse events reported with GLP-1 RA and insulin use varied across organ systems and showed no unique pattern based on type of GLP-1 RA or insulin therapy. Deaths and serious adverse events (events that were life-threatening or led to hospitalization) were rarely reported. In clinical trials, where comparators were included and event collection was most rigorous, the number of serious events was similar between patients treated with exenatide BID 52, lixisenatide QD 53 and placebo when each treatment was added to an existing basal insulin regimen.

### Hypoglycaemia

A major concern with antidiabetes treatments, particularly insulin and insulin secretagogues such as sulphonylureas or meglitinides, is the risk for iatrogenic hypoglycaemia. In contrast, GLP-1 RAs, when used without insulin secretagogues do not increase the risk for hypoglycaemia because of the glucose-dependent nature of their stimulation of insulin release (e.g. 23). The potential for GLP-1 RAs to lower glucose concentrations without increasing the risk for hypoglycaemia when used in combination with insulin is one of the regimen's most attractive features. Thus, the rate and incidence of hypoglycaemia across clinical trials and observational/clinical practice studies was explored (Table 4). While information on hypoglycaemic events was reported in many publications, the definitions used for classifying events were often not available. When definitions were provided, they differed across studies. For example, Riddle et al. defined mild hypoglycaemia as symptoms only or a blood glucose <3.9 mmol/l and moderate hypoglycaemia as a blood glucose <2.8 mmol/l 54. Buse et al. defined hypoglycaemia as signs or symptoms of hypoglycaemia with a blood glucose <3 mmol/l 52. Viswanathan et al. defined hypoglycaemia as blood glucose <3.4 mmol/l 42. While definitions for severe or major hypoglycaemia also differed across studies, they consistently included the aspect of requiring assistance for treatment. Given the diversity of definitions for hypoglycaemia, the most meaningful comparisons and patterns were discerned versus comparator treatments within clinical trials.

As with the efficacy results, information on hypoglycaemia from observational studies was available primarily for combinations of insulin with exenatide BID, consistent with the greater length of time exenatide has been commercially available compared to liraglutide. Among the observational trials which reported hypoglycaemia rates 16,41,43,45,46,62, between 2 and 12% of patients experienced hypoglycaemia while on the combination of exenatide BID and insulin. Severe hypoglycaemia was reported in 0–2% of patients in these trials.

Differences in designs and the availability of data in observational studies preclude comparison of results between the trials; however, several of these real-world studies provide useful internal comparisons. In the largest study (ABCD) Thong et al. 43 reported an 8.9% incidence of hypoglycaemia among patients who used exenatide BID in combination with insulin and OAMs compared with a 6.1% incidence among those using exenatide BID without insulin. There were 2 cases of severe hypoglycaemia (approximately 0.1% of patients) in the insulin/exenatide combination group and no cases reported in those using exenatide without insulin. There was no insulin only comparison group. A large insurance claims database (HealthCore Integrated Research database) review of patients who had filled prescriptions for exenatide BID and basal insulin examined hypoglycaemic events for those continuing concomitant use of both drugs compared to those who continued basal insulin alone or exenatide alone 62. The average number of hypoglycaemic events in a 6-month period was significantly lower among patients who continued basal insulin with exenatide (0.03 events/patient, 1.9% of patients) compared to those who continued on basal insulin without exenatide (0.1 events/patient, 2.9% of patients). Those who continued exenatide BID but stopped insulin experienced an average of 0.02 events/patient (1.8% of patients). To examine treatment sequence effects, Levin et al. compared efficacy and adverse events associated with exenatide BID added to insulin glargine and vice versa in two separate observational studies 45,46. In a retrospective chart review of 165 patients, Levin et al. 46 observed similar incidences of hypoglycaemia, mean glucose values and severe hypoglycaemia for both sequences. In an insurance claims database review of 422 patients, Levin et al. 45,63 reported an increase from 0.25 to 0.75 events per patient year for patients adding insulin glargine to exenatide BID while the rate increased from 0.17 to 0.57 events per patient year for those adding exenatide BID to insulin glargine. These data suggest, at least for exenatide BID, that the treatment sequence used was not associated with a difference in risk of hypoglycaemia.

As expected because of the more structured and rigorous collection of adverse events, the incidence of hypoglycaemia was greater in the randomized clinical trials compared to the observational studies. Buse et al. observed a 25% incidence of mild hypoglycaemia and 1.4 events/patient year among patients who added exenatide BID to insulin glargine compared to a 29% incidence and 1.2 events/patient year for patients who added placebo to existing insulin glargine 52. Riddle et al. reported a 53% incidence of mild hypoglycaemia with exenatide BID and insulin glargine compared to a 41% among patients receiving placebo and insulin glargine 54. Importantly, Buse et al. 52 and Riddle et al. 54 studied patients who were not using concomitant sulphonylurea which have previously been shown to increase the risk of hypoglycaemia when used in conjunction with exenatide. Seino et al. 53 examined a more diverse population of OAM users. Among patients not using a sulphonylurea, they reported a hypoglycaemia incidence of 33% when lixisenatide QD was added to basal insulin compared with 28% for placebo and basal insulin. When treatments were added to basal insulin plus sulphonylurea, the hypoglycaemia incidence was 47% among lixisenatide users while it remained 22% among placebo users 53. For these comparator-controlled studies, severe hypoglycaemia was rare with only Buse et al. 52 reporting events (<1% in placebo group, none with exenatide BID). These observations are consistent with those for patients treated with GLP-1 RAs in combination with metformin and/or thiazolidinediones in that addition of exenatide BID to a treatment regimen without insulin-secretagogues is not associated with an increased risk of hypoglycaemia 18,19,21. No data are available for randomized controlled studies comparing liraglutide QD in combination with basal insulin versus other treatment combinations including insulin.

An important consideration for evaluating hypoglycaemia risk associated with GLP-1 RA and insulin combination treatment is whether doses of existing medications are adjusted when GLP-1 RA or basal insulin is added to the other treatment. Details of dose adjustments were not available for most of the studies reviewed. Two studies described the insulin dose adjustments made when exenatide BID was added to existing insulin therapy. At treatment onset, Buse et al. 52 reduced insulin glargine doses by 20% in patients whose HbA1c was ≤8%. Titration of insulin glargine to glycaemic target values was not resumed in these patients until week 5 of treatment. In their observational study, Viswanathan et al. 42 noted that insulin doses were reduced by 10% if HbA1c was <7.5% at the time exenatide BID was added. Sulphonylurea use was discontinued in some studies 47,59,64. The variety of dose-adjustment scenarios across studies in addition to the differences in study designs complicates interpreting the value of prospective dose adjustments to hypoglycaemia risk.

### Potential Adverse Events of Special Concern

#### Acute Pancreatitis

Patients with type 2 diabetes have an increased risk of developing acute pancreatitis 65, a rare and potentially serious clinical event that has been observed with GLP-1 RA treatment in liraglutide QD clinical trials 66 and through post-marketing surveillance of marketed exenatide BID 33,67. Post-marketing surveillance is subject to reporting bias and cannot be used to calculate the incidence of an adverse event. In contrast, large epidemiological studies using different databases and analytic methods did not find a significantly increased risk of pancreatitis in users of exenatide BID compared with users of other antidiabetes agents 68–71, suggesting that incidence of pancreatitis may be related to the underlying disease rather that use of a particular drug. Among the studies of GLP-1 RA use in combination with insulin reviewed herein, Phillips et al. 48 reported two suspected cases of pancreatitis among patients who discontinued treatment due to abdominal pain with elevated amylase and lipase. Yoon et al. 41 also reported 1 case of pancreatitis that was confirmed by elevated serum lipase and computerized tomography scan of the pancreas. In the randomized controlled trials reviewed there were no reported cases of pancreatitis in patients treated with the GLP-1 RA and insulin combinations.

#### Cancer

Subjects with type 2 diabetes are at significantly higher risk for many forms of cancer and it has been hypothesized that chronic insulin treatment might facilitate neoplastic growth 72,73. An increased risk of thyroid tumours has been identified in nonclinical toxicology studies of GLP-1 RAs in rodents 33,66, however, the relevance of these findings to humans is unclear. One study analyzing the US FDA Adverse Events Reporting System (AERS) database showed a significantly increased reporting rate of pancreatic cancer in patients treated with GLP-1 based therapies (exenatide BID and sitagliptin) but the well-known limitations of the AERS database, including incomplete data and reporting biases preclude any conclusions from being drawn 74. Again, the reporting of cancer cases may be linked with the increase risk of cancer in patients with diabetes rather than treatment with a particular medication.

As both insulin analogues and GLP-1 RAs have been potentially associated with neoplasms, the question arises whether combined use of these agents would promote tumorigenicity. Evidence from *in vitro* and *in vivo* studies suggests that stimulation of insulin and GLP-1 receptors can cause cell proliferation via distinct intracellular signalling pathways. Whether there are additive or synergistic interactions is unknown; however, the potential proliferative effects of these pathways seem to be limited to specific types of cells. In the combination therapy literature reviewed herein one case of cancer (target tissue not specified) was reported in a retrospective observational study of exenatide BID used in combination with insulin in a study of obese patients with a long duration of diabetes (average 15.1 years) 16. No other tumours or malignancies were reported in the literature for the combination treatment. Due to the rarity of such adverse events, it would take much larger exposure to evaluate any potential additive relationship on cancer risk between GLP-1 RAs and insulin.

## Discussion

The wide variety of studies reviewed here provides a comprehensive assessment of combination therapy with GLP-1 RAs and basal insulin to improve glycaemic control and reduce body weight with a low risk of hypoglycaemia. The observational and clinical practice studies discussed tended to show greater weight loss than the randomized controlled trials while the randomized controlled trials tended to show greater glycaemic improvements with the combinations of GLP-1 RAs and basal insulin. Differences in how insulin doses were adjusted during the combination therapy may be a primary reason for the range of findings between studies. In general, for studies in which insulin was aggressively titrated to optimize glycaemic benefits, there was less weight benefit of the combination therapy. In contrast those studies with a protocol focus on insulin sparing tended to show greater weight loss and more modest glycaemic benefits with the combination therapy. In this context clinicians should avoid over-reduction of insulin and other diabetes medications when initiating GLP-1 RA combination therapy in poorly controlled patients (as discussed 43).

Overweight and obesity are prevalent in type 2 diabetes and the ability to offer patients therapies associated with weight loss are consistent with recent guidelines on ‘patient-centred’ or ‘individualized management’ approaches and can lead to improved medication adherence and patient satisfaction. These GLP-1 RA/basal insulin combinations may be particularly advantageous for obese patients with long-standing type 2 diabetes for mitigating the weight gain associated with insulin therapy, improving glycaemic control, and/or reducing insulin requirements. For patients who are unable to achieve adequate glycaemic control with basal insulin and OAMs, intensification of therapy with the addition of a short-acting GLP-1 RA may offer a number of advantages compared to the addition of rapid-acting prandial insulin. These include reduced risk of hypoglycaemia and weight gain compared to rapid-acting insulin 30. Obese patients also have elevated cardiovascular risk and importantly a number of the studies found that GLP-1 RA/insulin combination therapy was associated with improvements in cardiovascular risk factors, most notably systolic blood pressure and triglycerides. However, outcome studies are needed to measure the long-term effects of these combinations on cardiovascular morbidity and several such large studies are in progress which include combinations of the GLP-1 RAs and insulin (the liraglutide LEADER trial [NCT01179048], the exenatide EXSCEL trial [NCT01144338], and the dulaglutide REWIND trial [NCT01394952]; see http://www.clinicaltrials.gov).

Weight loss or attenuation of weight gain can be beneficial for slowing disease progression in type 2 diabetes and reducing mortality related to cardiovascular risk. In general, the addition of GLP-1 RAs to existing basal insulin regimens was associated with weight loss. In contrast, the addition of basal insulin to GLP-1 RAs was associated with no change in weight or an attenuation of the weight gain often observed in patients who have basal insulin added. This was shown in three controlled clinical trials examining the addition of basal insulin (glargine, detemir or ILPS) to existing GLP1-RA therapy 54–56,58,59. Despite the varied trial designs each combination resulted in reductions in HbA1c without significant weight gain.

To control the considerable health economic burden of diabetes, patient management must address obesity, cardiovascular disease, microvascular complications, renal complications and hypoglycaemia in considering the cost effectiveness of available treatment options. The issue of cost effectiveness is particularly important for GLP-1 RA/insulin combinations since the only insulins currently approved for this use are the relatively expensive long-acting analogues and GLP-1 RAs are also an expensive class of medications. However, because combination therapy with GLP-1 RAs and basal insulin improves glycaemic control, reduces body weight and improves cardiovascular risk factors it may lead to reduction in costly complications of diabetes. Indeed, a formal analysis of the cost-effectiveness of exenatide BID/insulin glargine conducted for the Scottish Medicines Consortium 75 using the extensively validated Centre for Outcomes Research Diabetes Model 76 and data estimated from Buse et al. 52 found a cost per quality-adjusted life year gained which meets thresholds considered cost-effective in the United Kingdom and the United States 77.

The majority of studies reviewed employed a basal, long-acting insulin, usually insulin glargine, for the combination. A few studies evaluated GLP-1 RA use in combination with multiple daily injection regimens of short-acting, rapid-acting or insulin mixtures. There is a compelling rationale for combining basal insulin (driving fasting glucose reduction) and a short-acting GLP-1 RA (driving postprandial glucose reduction due to effects on gastric emptying, glucagon and insulin) so from a clinical standpoint it makes more sense to substitute a GLP-1 RA for prandial insulin rather than combine the two agents. In this context, several studies reported the addition of exenatide BID was associated with a notable reduction of the rapid-acting insulin dose 16,41,42 or discontinuation of the insulin altogether 41. The merits of intensification of basal insulin therapy with a GLP-1 RA versus a prandial insulin are being directly investigated in two ongoing trials comparing the rapid-acting insulin lispro with either exenatide BID or the developmental agent albiglutide QW (Table 5).

**Table 5:** Ongoing and recently completed GLP1-RA + insulin studies from Clinicaltrials.gov

Citation	Phase	Intervention	Background treatment	Estimated enrollment	Treatment duration	Primary outcome	Status	Est. Completion for primary outcome
NCT01191268	III	Dulaglutide QW + lispro*	± OAMs	837	52 weeks	ΔHbA1c at 26 weeks	Active	February 2012
		Glargine + lispro*						
NCT00976391	III	Albiglutide QW + glargine	± OAMs	500	26 weeks	ΔHbA1c at 26 weeks	Completed	March 2011
		Lispro* + glargine						
NCT01476475	II	Lixisenatide QD + Glargine	Metformin	310	24 weeks	ΔHbA1c at 24 weeks	Recruiting	December 2012
		Glargine						
NCT00715624	III	Lixisenatide QD	Basal Insulin ± Metformin	450	24 weeks	ΔHbA1c at 24 weeks	Completed	February 2011
		Placebo						
NCT00975286	III	Lixisenatide QD	Glargine + Metformin ± TZD	446	24 weeks	ΔHbA1c at 24 weeks	Completed	August 2011
		Placebo						
NCT01336023	III	Liraglutide QD	OAMs	1660	26 weeks	ΔHbA1c at 26 weeks	Active	November 2012
		Degludec†						
		Liraglutide QD + degludec†						
NCT01392573	III	Degludec†	Metformin	382	26 weeks	ΔHbA1c at 26 weeks	Active	October 2012
		Liraglutide QD + Degludec†						
NCT01505673	IV	Liraglutide QD	High dose insulin	80	6 months	ΔHbA1c at 2 and 6 months	Recruiting	June 2013
		Placebo						
NCT00960661	III	Exenatide BID	Glargine Metformin ± SU	975	30 weeks	ΔHbA1c at 30 weeks	Recruiting	August 2012
		Lispro*						
NCT01006889	IV	Exenatide BID	Detemir	24	6 months	Hepatic steatosis	Active	December 2009
NCT01140893	II/III	Exenatide BID	CSII	110	26 weeks	ΔHbA1c at 26 weeks	Recruiting	May 2012
		Placebo						
NCT01076842	IV	Detemir	≥2 OAMs	75	24 weeks	ΔHbA1c at 24 weeks	Completed	April 2011
		Exenatide BID						
		Detemir + exenatide BID						

BID, twice daily; CSII, continuous subcutaneous insulin infusion; OAM, oral antihyperglycaemic medication; QD, once daily; QW, once weekly; SU, sulphonylurea; TZD, thiazolidinediones. Compiled from http://www.clinical trials.gov. Accessed 21 March 2012.

* Insulin lispro, a rapid-acting insulin.

† Insulin degludec, an long-acting insulin under development.

## Place in Therapy

Addition of a short-acting GLP-1 RA is a more convenient intensification strategy compared to adding meal-time injections of rapid-acting insulin because the fixed dosing does not require adjustments for meal sizes and carbohydrate content. These advantages may lead to greater compliance and patient satisfaction and future studies should address this possibility. Most of the literature reviewed herein reports studies of GLP-1 RA and insulin combination in patients with very advanced disease, consistent with the available treatment algorithms. More studies are needed to establish which other patients may benefit. For example, future studies should examine these combinations in patients with shorter duration of diabetes.

The studies reviewed were based predominantly on exenatide BID because of its tenure in the marketplace and there are fewer reports of liraglutide QD or lixisenatide QD used in combination with insulin. While the majority of benefits of GLP-1 RA can theoretically be exerted by all GLP-1 RAs, exenatide BID appears to exert a greater reduction on postprandial glucose compared to the longer-acting liraglutide QD 17 or exenatide QW 15. This distinction may be related to its shorter half-life and the peaking of its plasma concentration during the postprandial period and it suggests a potential niche for exenatide BID in combination with basal insulin 30. In contrast the longer-acting GLP-1 RAs exert greater effects on fasting glucose than the short-acting GLP-1 RAs and the selection of which agent to use among the available GLP-1 RAs can be guided, at least partially, by which glycaemic disturbance is more prominent in an individual patient 78. Head-to-head studies of combinations with insulin are needed to directly compare the relative merits of the various GLP-1 therapies.

There is no information available on combined use of the long-acting exenatide QW with insulin. However, the combination of a long-acting GLP-1 RA agonist with a short-acting insulin would also offer the advantages of complementary pharmacologies and could theoretically result in improvements of both fasting and postprandial glycaemic control. There are ongoing studies (Table 5) with the once weekly GLP-1 RAs in development (dulaglutide and albiglutide) with either basal or mealtime insulin.
